# Surface reconstruction and band bending in hydrogen-adsorbed $\hbox {Bi}_2\hbox {Se}_3$ topological insulator

**DOI:** 10.1038/s41598-020-71398-9

**Published:** 2020-09-02

**Authors:** Kyu Won Lee, Cheol Eui Lee

**Affiliations:** Department of Physics, Korea University, Seoul, 02841 Republic of Korea

**Keywords:** Condensed-matter physics, Nanoscale materials

## Abstract

We have investigated the effects of hydrogen adsorption on the $\hbox {Bi}_2\hbox {Se}_3$ topological insulator by using the density functional theory calculations. We found that hydrogen adsorption on the surface leads to surface reconstruction to reduce the band bending effect. Contrasting to a previous report that hydrogen adsorption transforms the single Dirac cone at the Brillouin zone center into three Dirac cones at the zone boundary, the Dirac cones at the zone center corresponding to the topological surface states were confirmed to be robust against the hydrogen adsorption and surface reconstruction. Hydrogen adsorption induces a Rashba-like spin-splitting of the topological surface state, and can introduce Rashba-like quantum well states within the bulk gap, which can be attributed to a semiconductor-like band bending at an interface.

## Introduction

$\hbox {Bi}_2\hbox {Se}_3$ is a representative and prototypical three-dimensional topological insulator, as confirmed theoretically and experimentally^1,2^. Topological insulators are characterized by metallic states at the surface of bulk insulators, which are protected by time reversal symmetry and spin-orbit coupling (SOC)-induced bulk band gap^3^. The surface metallic states exhibit linear energy dispersion forming a Dirac cone within the bulk band gap, and are free from backscattering due to the nonmagnetic impurities^3^. While the topological surface states are robust against surface perturbations, the details of the surface states have been reported to strongly depend on the surface condition, showing a shift of the Dirac point and the Rashba-like surface quantum well states^4–8^.

A near surface electrostatic potential, which results in a semiconductor-like band bending, has been considered to be responsible for the details of the surface states^9–11^. Potassium adsorption and selenium vacancies on the surface of $\hbox {Bi}_2\hbox {Se}_3$ were reported to give rise to a long-ranged potential perpendicular to the surface, resulting in the band bending effect^12^. Chromium adsorption on the surface was reported to induce the Rashba-like surface states without causing a gap opening at the Dirac point, contrasting to bulk doping of chromium^13,14^. Iodine adsorption on the surface was reported to induce additional Dirac cones at the zone center that cannot be attributed to the Rashba effect and was attributed to the proximity effect in the case of weak hybridization^15^.

Shockley criterion has been introduced to describe the details of the topological surface states with different surface terminations, where the phase shift for wavefunction matching changes and the surface bands move sideways^16^. Hydrogen adsorption was reported to transform the Dirac cone at the Brillouin zone center corresponding to the H-adsorbed surface into three Dirac cones at the zone boundary, which was described by the Shockley criterion^16,17^. In other works, the single Dirac cone at the zone center within the bulk gap was described by a band bending^9,11,12^. Due to the adsorption-induced potential, the Dirac cone corresponding to the adsorption surface shifts down into the valence bands, leaving a single Dirac cone corresponding to the pristine surface within the bulk gap^9,11,12^.

Recently, hydrogen adsorption on ZnSe-covered $\hbox {Bi}_2\hbox {Se}_3$ has been investigated for hydrogen storage, and hydrogen adsorption on $\hbox {Bi}_2\hbox {Se}_3$ has been proposed as a possible mechanism to cause surface conduction electron spin resonance signals^18,19^. In this work, we have investigated the effects of hydrogen adsorption on the topological insulator $\hbox {Bi}_2\hbox {Se}_3$ by using the density functional theory (DFT) calculations. We found that hydrogen adsorption on the surface leads to surface reconstruction to reduce the band bending effect. The two Dirac cones at the Brillouin zone center corresponding to the topological surface states were confirmed to be preserved regardless of the surface adsorption and reconstruction. It was revealed that the Dirac cone at the zone boundary is a surface state but not a topological surface state, contrasting to previous works^16,17^. Hydrogen adsorption at the van der Waals spacing introduces a new interface state within the bulk gap, which can be attributed to the Rashba-like quantum well state due to the band bending at the interface.

## Results and discussion

**Figure 1:**
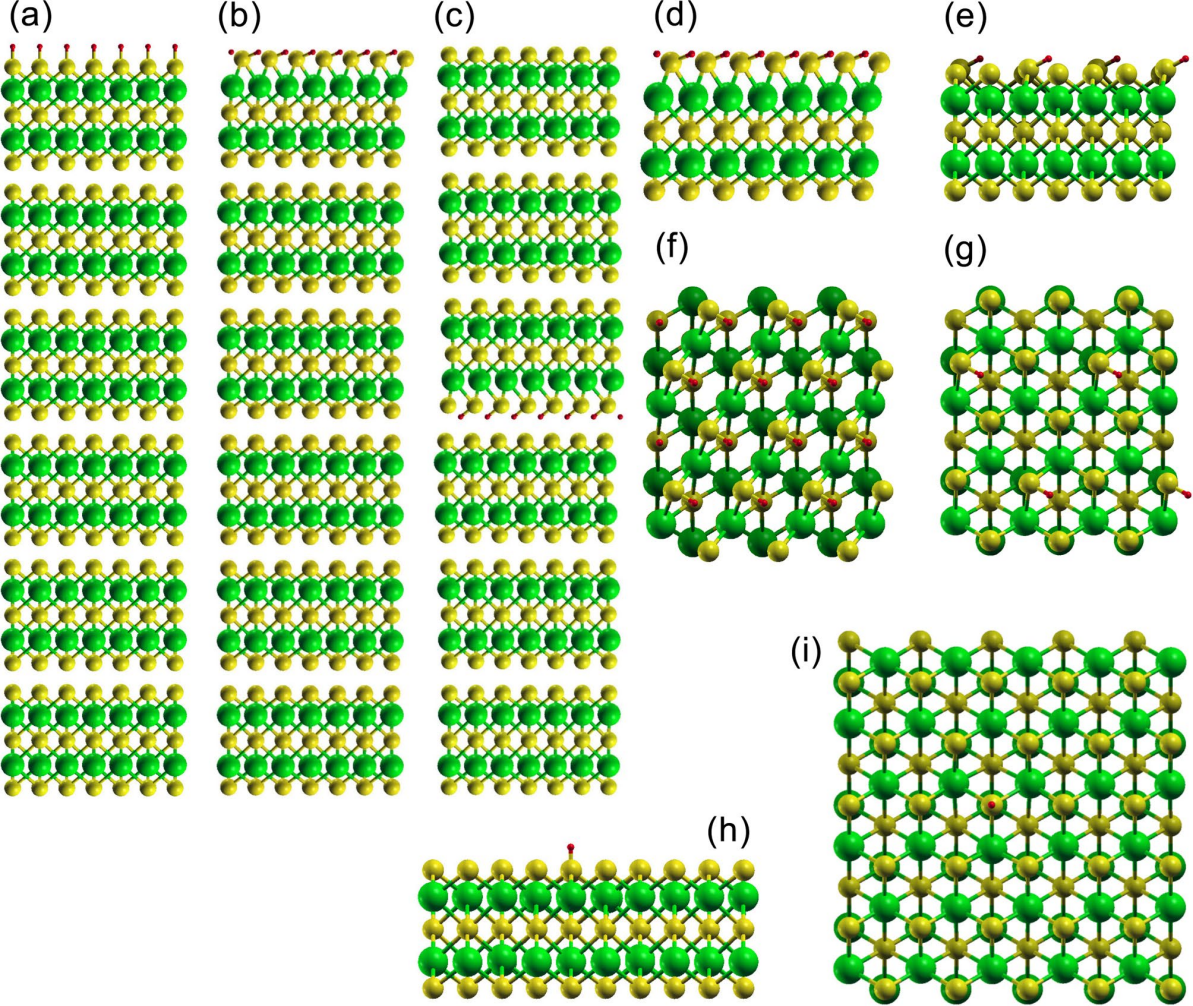
Structure of H-adsorbed $\hbox {Bi}_2\hbox {Se}_3$ slabs. Yellow, green and red balls correspond to Se, Bi and H atoms, respectively. (**a**) Side view of 6-QL slab with H only on the top layer and not on the bottom layer, where only the H position was relaxed. (**b**) Side view of fully-relaxed 6-QL slab with H only on the top layer and not on the bottom layer. (**c**) Fully-relaxed 6-QL slab with H only at the vdW spacing in the middle of the slab and not on the outer surface. (**d**–**i**) Side and top views of fully-relaxed 1-QL slab with H only on the top layer and not on the bottom layer with different H coverage. (**d**,**f**) One H per unit cell as shown in (**b**). (**e**,**g**) one H per $2 \times 2$ supercell. (**h**,**i**) One H per $4 \times 4$ supercell.

$\hbox {Bi}_2\hbox {Se}_3$ has a rhombohedral crystal structure belonging to the spatial group ${R{\bar{3}}m}$^2^. $\hbox {Bi}_2\hbox {Se}_3$ consists of five strongly-bonded atoms Se–Bi–Se–Bi–Se, forming a quintuple layer (QL), and the QLs are weakly bonded by van der Waals (vdW) interactions. Figure 1 shows the structure of hydrogen-adsorbed $\hbox {Bi}_2\hbox {Se}_3$ slabs, Fig. 1a showing 6-QL slab with H only on the top layer and not on the bottom layer, where only the H position was relaxed. The H atom is located just above the outer Se atom and the bond length of H–Se was obtained to be 0.155 nm. Figure 1b show the fully-relaxed structure of 6-QL slab with H only on the top layer and not on the bottom layer, where the bond length of H–Se was obtained to be 0.152 nm. Figure 1c shows the fully-relaxed structure of 6-QL slab with H only at the vdW spacing in the middle of the slab and not on the outer surface, where the vdW spacing increased by about 0.06 nm.

Figure 1d–i show the side and top views of fully-relaxed 1-QL slab with H only on the top layer and not on the bottom layer with different H coverages. Figure 1d,f correspond to one H per unit cell as shown in Fig. 1b. Hydrogen adsorption induces a surface reconstruction as shown in Fig. 1d,f. The outer Se atom of a QL has three Se–Bi bonds, and hydrogen adsorption on the Se atom breaks one of the three Se–Bi bonds. Surface reconstruction occurs in 1-QL slab in the same way as in 6-QL slab, which is because the QLs are weakly bonded by vdW interactions. Comparing Fig. 1b,d, we can confirm that the hydrogen-adsorbed surface has the same structure. Thus, the fully-relaxed 1-QL slab was used to investigate surface reconstruction at low H coverages, reducing the computation time required. Figure 1e,g correspond to one H per $2 \times 2$ supercell, where H adsorption still breaks one of the three Se–Bi bonds but surface reconstruction is weakened. The bond length of H–Se was obtained to be 0.152 nm. Figure 1h,i correspond to one H per $4 \times 4$ supercell, where the bond length of H–Se was obtained to be 0.150 nm. At these low H coverages, the three Se–Bi bonds are maintained and the H atom is located just above the outer Se atom like when only the H position was relaxed. As a result, the surface reconstruction induced by H adsorption depends on the H coverage.

The total energy per $\hbox {Bi}_2\hbox {Se}_3$ unit ${\Delta E_{tot}}$ was calculated with respect to the fully-relaxed 6-QL slab with H only on the top layer as shown in Fig. 1b. When only the H position in the 6-QL slab with H only on the top layer was relaxed as shown in Fig. 1a, ${\Delta E_{tot}}$ was + 0.216 eV indicating that the reconstructed surface is more stable than the unreconstructed surface. In the fully-relaxed 6-QL slab with H only at the vdW spacing in the middle of the slab as shown in Fig. 1c, ${\Delta E_{tot}}$ was $+$ 0.047 eV. In the fully-relaxed 6-QL slab with H adsorbed only on the inner Se atom of the top QL (not shown), ${\Delta E_{tot}}$ was $+$ 0.141 eV. Hydrogen adsorption on the Bi atom was found to be unstable and, after full relaxation, hydrogen was adsorbed on the adjacent Se atom. The surface reconstruction in Fig. 1 appears to be the most stable one. The topological surface states are robust against surface perturbations, which is also true for the surface reconstruction as will be further confirmed in this work.

**Figure 2:**
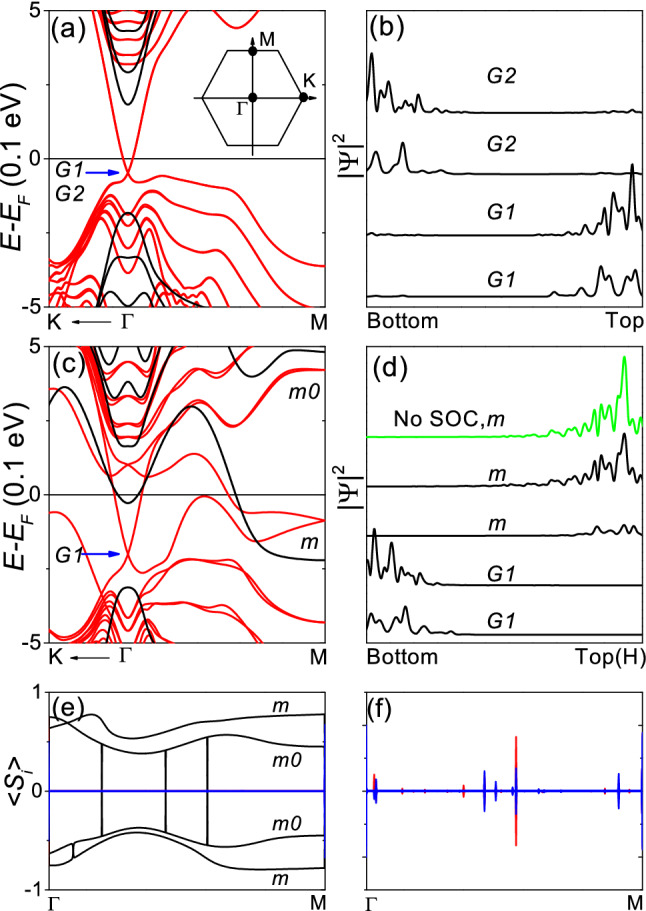
Electronic properties of only-H-relaxed 6-QL slab with H only on the top layer. (**a**,**b**) Pristine slab. (**a**) Band structure. The red and black lines correspond to the calculation with and without SOC, respectively. (**b**) Probability density ${|\Psi |^2}$. (**c**–**f**) Slab with H only on the top layer and not on the bottom layer, where only the H position was relaxed. (**c**) Band structure. (**d**) ${|\Psi |^2}$, where the green line corresponds to the calculation without SOC. (**e**) Spin expectation values of the states connected to *m* and ${m_0}$. The black, red and blue lines correspond to ${<S_{x}>}$, ${<S_{y}>}$ and ${<S_{z}>}$, respectively, in units of ${\hbar /2}$. (f) ${<S_{y}>}$ and ${<S_{z}>}$ of all the states within ${E=E_{F}\pm }\,1.5$ eV.

Figure 2a,b correspond to a pristine 6-QL slab, Fig. 2a showing the band structure. In the absence of SOC, the band structure has a gap. In the presence of SOC, topological surface states appear within the bulk gap, forming the Dirac cones at the ${\Gamma }$ point. The Dirac cones are doubly degenerate, corresponding to two equivalent surfaces as shown in Fig. 2b. Figure 2b shows the probability density ${|\Psi |^2}$ at the degenerate Dirac points *G*1 and *G*2 indicated in Fig. 2a.

Figure 2c–f correspond to 6-QL slab with H only on the top layer, where only the H position was relaxed, Fig. 2c showing the band structure. In the presence of SOC, the band structure has a single Dirac cone at the ${\Gamma }$ point and another Dirac cone at the M point within the bulk gap, consistently with a previous work^17^. The Dirac cones at the ${\Gamma }$ and M points correspond to the bottom and to the H-adsorbed top layers, respectively, as shown in Fig. 2d. Figure 2d shows ${|\Psi |^2}$ at the Dirac points *G*1 and *m* indicated in Fig. 2c. However, even in the absence of SOC, an adsorption-induced state already exists within the bulk gap at the M point as shown in Fig. 2c, and the adsorption-induced state is a surface state confined on the H-adsorbed top layer as shown in Fig. 2d using the green line. The surface state at the M point is not a topological surface state because $\hbox {Bi}_2\hbox {Se}_3$ is an ordinary band insulator in the absence of SOC.

Spin-momentum locking in the topological insulator allows symmetry-protected surface states^3^. In a previous work, the two surface states connecting the *G*1 and *m* points indicated in Fig. 2c were reported to be strictly spin-polarized along the direction perpendicular to the ${\Gamma }$-M line^17^. The spin polarization perpendicular to the ${\Gamma }$-M line was attributed to the spin-momentum locking and was presented as evidence that the Dirac cone at the M point corresponds to the topological surface states^17^. As shown in the inset of Fig. 2a, the ${\Gamma }$-M line is on the *y* axis in this work. Figure 2e shows the spin expectation values. As has been reported^17^, the two surface states connected to the *m* point are fully spin-polarized along the *x* axis with ${<S_{y}>=<S_{z}>=0}$. However, the two bulk states connected to the *m*0 point indicated in Fig. 2c are also fully spin-polarized along the *x* axis. Figure 2f shows ${<S_{y}>}$ and ${<S_{z}>}$ of all the states within ${E=E_{F}\pm }$ 1.5 eV, where ${<S_{y}>}$ and ${<S_{z}>}$ are zero for almost all the states. The spin polarization cannot be attributed to a topological surface state. As discussed immediately below, the band bending effect in the unrelaxed structure is much stronger than that in the relaxed structure, which may be responsible for the spin polarization.

**Figure 3:**
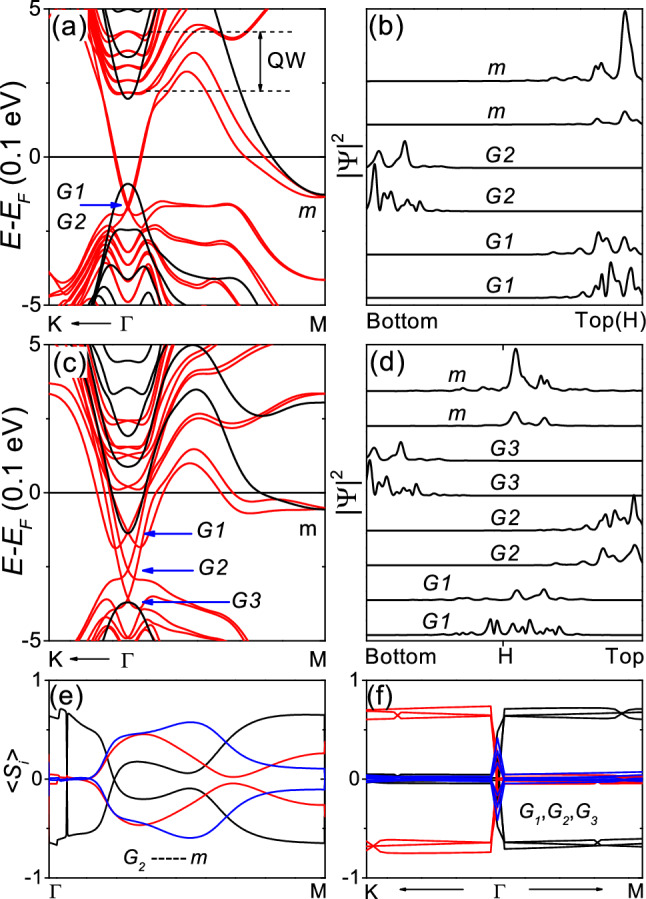
Electronic properties of fully-relaxed 6-QL slab with H adsorbed. (**a**,**b**) Fully-relaxed slab with H only on the top layer and not on the bottom layer. (**a**) Band structure. The red and black lines correspond to the calculation with and without SOC, respectively. (**b**) ${|\Psi |^2}$. (**c**–**f**) Fully-relaxed slab with H only at the vdW spacing in the middle of the slab and not on the outer surface. (**c**) Band structure. (**d**) ${|\Psi |^2}$. Spin expectation values (**e**) of the two surface states connecting *G*2 and *m* along the ${\Gamma }$-M line and (**f**) of the Dirac cones (*G*1, *G*2 and *G*3) near the ${\Gamma }$ point. The black, red and blue lines correspond to ${<S_{x}>}$, ${<S_{y}>}$ and ${<S_{z}>}$, respectively, in units of ${\hbar /2}$.

Figure 3a,b show the band structure and ${|\Psi |^2}$ of fully-relaxed 6-QL slab with H only on the top layer, respectively. In the presence of SOC, the band structure has two Dirac cones with a slight Rashba-like splitting at the ${\Gamma }$ point and a band crossing at the M point within the bulk gap, as shown in Fig. 3a. As shown in Fig. 3b, the two Dirac cones correspond to the top and bottom layers, and the band crossing at the M point corresponds to surface states confined at the H-adsorbed top layer. The surface states at the M point already exist even in the absence of SOC and did not show spin-momentum locking, indicating that the surface states are not topological ones. The Rashba-like band structure with a small downshift of the Dirac point can be attributed to the adsorption-induced band bending at the vacuum interface. Considering the small Rashba splitting and downshift of the the Dirac point, the adsorption-induced surface reconstruction appears to reduce the band bending effect.

Figure 3c,d show the band structure and ${|\Psi |^2}$ of fully-relaxed 6-QL slab with H only at the vdW spacing in the middle of the slab, respectively. In the presence of SOC, the band structure has three Dirac cones at the ${\Gamma }$ point and a band crossing at the M point within the bulk gap, as shown in Fig. 3c. As shown in Fig. 3d, the two Dirac cones at *G*2 and *G*3 correspond to the topological surface states confined at the top and bottom layers, respectively. The crossing bands at *G*1 and *m* are interface states confined near the hydrogen-adsorbed layer. Thus, the 6-QL slab with H only at the vdW spacing in the middle of the slab can be considered as a junction between the pristine 3-QL slab and the H-adsorbed 3-QL slab, both of which slabs are topological insulators.

The interface states at the M point already exist even in the absence of SOC as shown in Fig. 3d, and did not show spin-momentum locking as shown in Fig. 3e, indicating the the interface states are not topological surface states. As shown in Fig. 3f, the interface states (*G*1) at the ${\Gamma }$ point as well as the surface states (*G*2 and *G*3) show spin-momentum locking. Because the interface between the same topological insulators cannot have topological interface states, the interface states (*G*1) at the ${\Gamma }$ point can be attributed to the band bending at the junction. Comparing Figs. 2a and 3a, the Fermi level is higher in the hydrogen-adsorbed slab than in the pristine slab. Compared to Fig. 3a, the Rashba-split quantum well (QW) states shift to lower energy in Fig. 3c, and the lowest quantum well state appears to correspond to the interface state *G*1.

**Figure 4:**
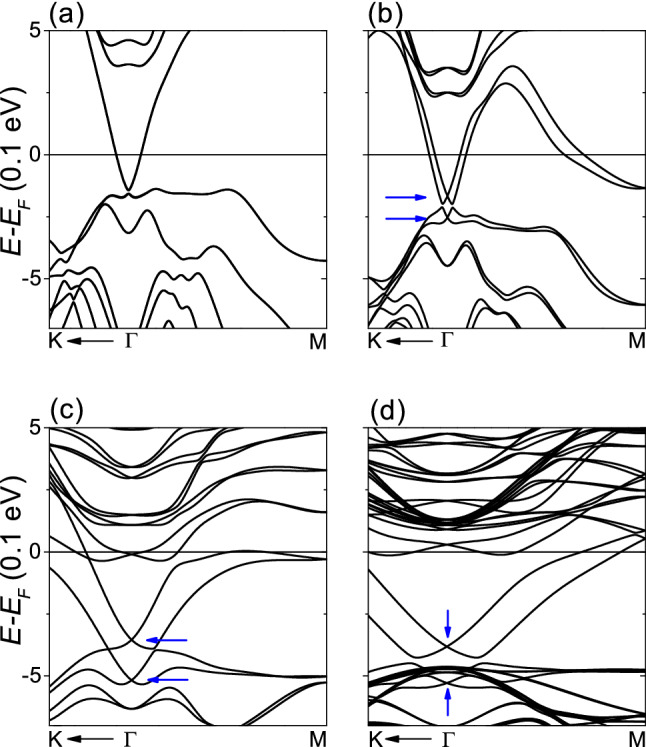
Fully-relaxed 3-QL slab with H only on the top layer. (**a**) Pristine slab. (**b**) One H per unit cell. (**c**) One H per ${2\times 2}$ supercell. (**d**) One H per ${4\times 4}$ supercell. Blue arrows indicate the Dirac points.

As shown in Fig. 1, the surface reconstruction induced by H adsorption is weakened at low H coverages. For one H per $4 \times 4$ supercell, the three Se–Bi bonds are maintained and the H atom is located just above the outer Se atom like when only the H position was relaxed. We tried to make sure that the electronic properties of a fully-relaxed slab with low H coverage is similar to when only the H position was relaxed. Figure 4 shows the band structures of fully-relaxed 3-QL slab with H only on the top layer for different H coverages. For one H per $4\times 4$ supercell, only the top QL on which hydrogen was adsorbed was relaxed. In Fig. 4a corresponding to the pristine 3-QL slab, the topological surface states are well observed, although there is a small gap. Thus, the fully-relaxed 3-QL slab was used to investigate the electronic properties at low H coverages, reducing the computation time required. As shown in Fig. 4b–d, as the H coverage decreases, the Fermi level and Rashba splitting increase, and the surface state at the M point merges into the conduction band. And, we can see that the two Dirac cones at the ${\Gamma }$ point, corresponding to the topological surface states, are preserved regardless of surface modification and H coverage. As a result, a fully-relaxed slab with low H coverage may have a surface structure similar to when only the H position was relaxed, but the electronic properties are significantly different from when only the H position is relaxed.

To summarize, we have investigated the effects of hydrogen adsorption on the topological insulator $\hbox {Bi}_2\hbox {Se}_3$ by using the density functional theory calculations. Hydrogen adsorption leads to surface reconstruction to reduce the band bending effect. The two Dirac cones corresponding to the topological surface states are preserved regardless of the surface modification. In $\hbox {Bi}_2\hbox {Se}_3$ slab with hydrogen adsorbed at the van der Waals spacing, the interface states confined at the hydrogen-adsorbed layer appear within the bulk gap, which can be attributed to the Rashba-split quantum well states due to the band bending at the interface.

## Methods

A SIESTA package^20^, which uses a localized linear combination of numerical atomic-orbital basis sets, was employed for the DFT calculations. A generalized gradient approximation of Perdew-Burke-Ernzerhof was used for the exchange and correlation potential^21^. A double-${\zeta }$ polarized basis set and norm-conserving fully-relativistic pseudopotentials were used. The plane-wave cutoff energy of 200 Ry and *k*-points of $10 \times 10 \times 1$ meshes in a Monkhorst-Pack scheme were used. The lattice constant of $\hbox {Bi}_2\hbox {Se}_3$ was set to $a=0.4138$ nm and the DFT calculations were performed on the $\hbox {Bi}_2\hbox {Se}_3$ (111) slabs. If not specified, one hydrogen per unit cell was adsorbed on the slab. The atomic coordinates were optimized by using the conjugated gradients method with a maximum force tolerance of 0.1 eV/nm with the nonlocal van der Waals density functional of Dion et al. as implemented by Román-Pérez and Soler^22–24^. A vacuum spacing between the $\hbox {Bi}_2\hbox {Se}_3$ slabs was set to 2.0 nm.
